# Immunome Knowledge Base (IKB): An integrated service for immunome research

**DOI:** 10.1186/1471-2172-10-3

**Published:** 2009-01-09

**Authors:** Csaba Ortutay, Mauno Vihinen

**Affiliations:** 1 Institute of Medical Technology, FI-33014 University of Tampere, Finland; 2 Tampere University Hospital, FI-33520 Tampere, Finland

## Abstract

**Background:**

Functioning of the immune system requires the coordinated expression and action of many genes and proteins. With the emergence of high-throughput technologies, a great amount of molecular data is available for the genes and proteins of the immune system. However, these data are scattered into several databases and literature and therefore integration is needed.

**Description:**

The Immunome Knowledge Base (IKB) is a dedicated resource for immunological information. We identified and collected genes that are essential for the immunome. Nucleotide and protein sequences, as well as information about the related pseudogenes are available for 893 human essential immunome genes. To allow the study of the evolution of the immune system, data for the orthologs of human genes was collected. In addition to the human immunome, ortholog groups of 1811 metazoan immunity genes are available with information about the evidence of their immunity function. IKB combines three previous databases and several additional data items in an integrated system.

**Conclusion:**

IKB provides in one single service access to several databases and resources and contains plenty of new data about immune system. The most recent addition is variation data on genomic, transcriptomic and proteomic levels for all the immunome genes and proteins. In the future, more data will be added on the function of these genes. The service has a free and public web interface.

## Background

The human immune system is a very complex biological machinery in which hundreds of proteins are involved. Lots of data is available at the molecular, structural, cellular and organ levels, in both normal and diseased states. A comprehensive compilation and database of the human immune system was made recently [1,2]. The term 'immunome' is used to describe all the genes and proteins taking part in immune responses, excluding those that are widely expressed in cell types outside the immune system.

Immunology-related genes and their corresponding proteins were collected from research articles, textbooks, and electronic information sources for creating the immunome set. CD (cluster of differentiation) proteins for cell surface molecules are defined by the human leukocyte differentiation antigen (HLDA) workshops [3]. In addition to classical and alternative complement system, lectin pathway and the components of the membrane attack complex were included together with chemokines, cytokines, and their receptors.

Immunodeficiency-related entries were obtained from the ImmunoDeficiency Resource [4] and IDbases [5]. Immunology-related Gene Ontology [6] terms were utilized to identify genes involved in immunological processes. Genes involved in both innate and adaptive immunity were included. Immunome genes have to be essential for the immune system but not widely expressed in many non-immunological cells and tissues. Thus, only those proteasome components that play a role in the transformation to the immunoproteasome and in its regulation were included.

Vertebrate immunoglobulins, B and T cell receptors and major histocompatibility complex (MHC) members were excluded from the immunome because they are formed from gene fragments and thus do not represent complete genomic genes. Further, these are already well covered in the international ImMunoGeneTics information system (IMGT) [7] and IMGT/HLA database [8].

The immunome dataset serves many kinds of research ranging from evolutionary studies to systems biology, structural biology, and immunodeficiencies, etc. We have previously analyzed the human immunome genes and proteins and constructed a database for the Immunome [2], investigated the molecular evolution of the immunome and released for that purpose the ImmTree database [9]; and studied and identified orthologs for metazoan immunome genes and collected them in ImmunomeBase [10]. To allow studies in all these datasets it became necessary to integrate the individual registries. While doing so we updated the registries and added several new data items so that the new Immunome Knowledge Base (IKB) facilitates versatile and comprehensive studies of the immune system. As an example of the utilization of the integrated data, we recently found that the efficiency of the protein interaction network of the immunome increases during evolution [11], which is against the current paradigm for small world networks. Protein interaction network information and Gene Ontologies of immune genes allowed us to prioritize novel immunodeficiency candidate genes based on data in IKB [12].

## Construction and content

### Immunome, ImmTree and ImmunomeBase databases

**Figure 1:**
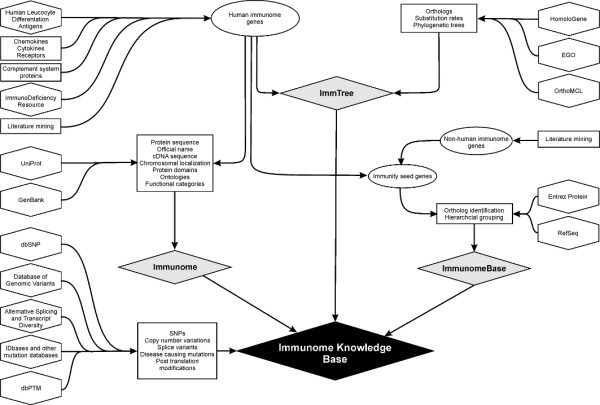
**Data content and integration in the Immunome Knowledge Base. Information from different sources and databases (indicated by boxes and polygons) was compiled originally in three individual databases (grey diamonds)**. These resources and new additional information were combined and integrated in IKB (black diamond).

The three previously released databases, Immunome, ImmTree and ImmunomeBase, contain different but related information. To allow a seamless combination of information from these resources they were integrated to the IKB (Fig. 1).

The immunome data is based on an exhaustive analysis of literature and databases, originally yielding 847 genes [1,2]. Because adaptive and innate immunity collectively include very large number of biological responses and proteins, the functions of immune proteins vary widely from cell surface recognition, transcription factors, and DNA processing to adaptor proteins, etc. Entries in the Immunome registry include cross references to UniProt and GenBank, official Human Gene Nomenclature Committee (HGNC) [13] names as well as alternative names, and information of location in chromosomes (Fig. 1). For functional annotation, Gene Ontology (GO) [6] terms are provided. In addition, we have manually classified the proteins based on the immunological processes in which they are involved.

ImmTree was developed to explore the molecular evolution of the immune system. Orthologous genes for those in the human immunome were identified from HomoloGene [14], EGO [15] and OrthoMCL [16]. In addition to information for sequences and substitution rates for human-mouse comparisons, there are multiple sequence alignments and phylogenetic trees, calculated with parsimony methods, available.

ImmunomeBase is a multi-species database of immunity that contains metazoan ortholog gene groups. Human immunome genes, along with others specific for some other organisms identified from literature and databases, were used as seeds in reciprocal BLAST searches against the non-redundant protein database. A two-level system was developed for the grouping of orthologs.

### Update and addition of new entries

Several new genes/proteins have been added to the integrated IKB. Originally there were 847 genes and now there are 893 in the human immunome dataset. Correspondingly, the number of data items have grown to 2954 multiple sequence alignments and phylogenetic trees and 1059 level 1 and 1147 level 2 ortholog groups for 1811 metazoan seed genes. Altogether 46 new genes were included and with the new variation data approximately 100000 new data items were added.

As new genes are identified and related to immunological processes the number of genes in IKB will grow in the future. IKB will be frequently updated.

## Utility and Discussion

The scope and coverage of IKB was expanded by new information. Genetic variations were integrated from the ImmunoDeficiency Mutation Databases (IDbases) [5], currently available for some 130 genes. These repositories contain over 5000 patient cases. Copy number variations (CNVs) were taken from the Database of Genomic Variants [17], in which information has been collected from several large-scale experiments. CNV data is currently available for 368 immunome genes.

Splice variants are from the Alternative Splicing and Transcript Diversity registry [18]. Altogether 3681 alternative forms for 495 human genes are included. Single nucleotide polymorphisms (SNPs) originate from dbSNP [19]. 54013 SNPs for 825 genes are included. Post-translational modifications, mainly phosphorylations, were taken from dbPTM [20], from where 2756 modifications for 394 gene products were included.

### Integration of the resources

**Figure 2:**
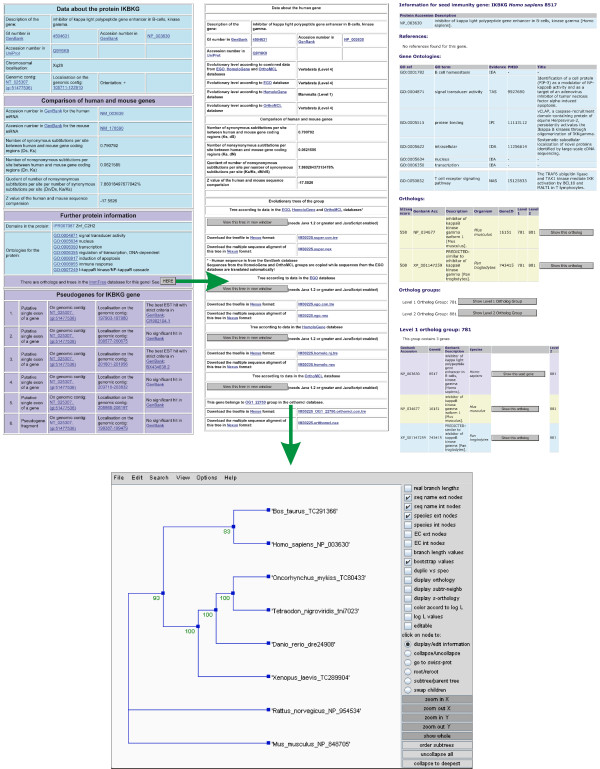
**An example of information in IKB**. Results for the gene IKBKG. Left, basic facts about the gene and the protein along with gene ontologies and related pseudogenes. A link points to the page containing orthologs and phylogenetic trees with multiple sequence alignments, shown in the middle. As this gene is a member of a bigger metazoan ortholog group there is data about the groups and group member proteins available to the right. Bottom, a detailed phylogenetic tree for the ortholog group.

The three earlier resources were integrated and new data was added to generate the IKB (Fig. 1). First, the Immunome and ImmTree databases were merged and crosslinked. Results for queries to immunome sequences now have pointers also to the evolutionary information and phylogenetic trees, whenever available. A new search engine was developed to cover the entire resource. An example of results for a IKBKG query is in Fig. 2. The results allow the user to easily compare and compile information from different sources.

IKB has been implemented as a relational database using a MySQL database engine. The search engine utilizes perl CGI scripts to provide many options for online users. Result pages contain, in addition to IKB information, links to primary databases as well. The user can retrieve pre-defined groups of genes from human immunome or even list all the available genes from IKB.

## Conclusions and perspectives

IKB is a comprehensive service providing information about genes and proteins involved in immunological processes, their evolutionary history, orthologous genes and genetic variations at many levels including SNPs, disease-causing mutations, alternatively spliced variants and copy number variations. In addition, variants at protein level, i.e. post-translational modifications are included. IKB combines three previously independent services and several new types of information within a single service that allows easy access and versatile queries across the data.

Some other databases address the immune system, but none with the scope and breadth of IKB. The Immuno Polymorphism Database (IPD) [21] hosts databases dedicated to human Killer-cell Immunoglobulin-like Receptors, MHC molecules, human platelet antigens and tumour cell lines. Information about immunoglobulins, T cell receptors, and the MHC system of human and other vertebrates, as well as some other related proteins, is available from the IMGT databases [7,8]. The gene fragments in these resources are excluded from IKB, which also has a strong emphasis on the evolution of the immune system, providing phylogenetic trees, ortholog groups and nucleotide substitution data in addition to variation information on DNA, RNA and protein levels.

Data in IKB can be used for large scale studies targeting immune systems. Since IKB contains immunity related functional categories and other auxiliary data with a broad scope, it can be used to place immunity related research results in context of other type of data. For example, results for disease related transcriptome studies can be analysed based on data in IKB such as ontology terms and evolutionary information. Evolution related information from IKB could be used to investigate how genes with important functions in diseases have emerged and how the functions have been conserved.

We are currently developing a system to automatically update the database. New types of data will be added in the near future, including protein-protein interactions of immunome members.

## Availability and requirements

IKB is freely available for academic research at .

## Authors' contributions

CO implemented the service using perl and MySQL. MV designed and coordinated the project. All authors drafted the manuscript and approved its content.

## References

[B1] Ortutay C, Vihinen M (2006). Immunome: a reference set of genes and proteins for systems biology of the human immune system. Cell Immunol.

[B2] Ortutay C, Siermala M, Vihinen M (2007). Molecular characterization of the immune system: emergence of proteins, processes, and domains. Immunogenetics.

[B3] Zola H, Swart B, Nicholson I, Aasted B, Bensussan A, Boumsell L, Buckley C, Clark G, Drbal K, Engel P, Hart D, Horejsi V, Isacke C, Macardle P, Malavasi F, Mason D, Olive D, Saalmueller A, Schlossman SF, Schwartz-Albiez R, Simmons P, Tedder TF, Uguccioni M, Warren H (2005). CD molecules 2005: human cell differentiation molecules. Blood.

[B4] Väliaho J, Pusa M, Ylinen T, Vihinen M (2002). IDR: the ImmunoDeficiency Resource. Nucleic Acids Res.

[B5] Piirilä H, Väliaho J, Vihinen M (2006). Immunodeficiency mutation databases (IDbases). Hum Mutat.

[B6] Ashburner M, Ball CA, Blake JA, Botstein D, Butler H, Cherry JM, Davis AP, Dolinski K, Dwight SS, Eppig JT, Harris MA, Hill DP, Issel-Tarver L, Kasarskis A, Lewis S, Matese JC, Richardson JE, Ringwald M, Rubin GM, Sherlock G (2000). Gene ontology: tool for the unification of biology. The Gene Ontology Consortium. Nat Genet.

[B7] Robinson J, Waller MJ, Parham P, de Groot N, Bontrop R, Kennedy LJ, Stoehr P, Marsh SG (2003). IMGT/HLA and IMGT/MHC: sequence databases for the study of the major histocompatibility complex. Nucleic Acids Res.

[B8] Lefranc MP, Giudicelli V, Kaas Q, Duprat E, Jabado-Michaloud J, Scaviner D, Ginestoux C, Clement O, Chaume D, Lefranc G (2005). IMGT, the international ImMunoGeneTics information system. Nucleic Acids Res.

[B9] Ortutay C, Siermala M, Vihinen M (2007). ImmTree: database of evolutionary relationships of genes and proteins in the human immune system. Immunome Res.

[B10] Rannikko K, Ortutay C, Vihinen M (2007). Immunity genes and their orthologs: a multi-species database. Int Immunol.

[B11] Ortutay C, Vihinen M (2008). Efficiency of the immunome protein interaction network increases during evolution. Immunome Res.

[B12] Ortutay C, Vihinen M Identification of candidate disease genes by integrating Gene Ontologies and protein interaction networks: Case study of primary immunodeficiencies. Nucleic Acids Res.

[B13] Eyre TA, Ducluzeau F, Sneddon TP, Povey S, Bruford EA, Lush MJ (2006). The HUGO Gene Nomenclature Database, 2006 updates. Nucleic Acids Res.

[B14] Wheeler DL, Barrett T, Benson DA, Bryant SH, Canese K, Chetvernin V, Church DM, Dicuccio M, Edgar R, Federhen S, Feolo M, Geer LY, Helmberg W, Kapustin Y, Khovayko O, Landsman D, Lipman DJ, Madden TL, Maglott DR, Miller V, Ostell J, Pruitt KD, Schuler GD, Shumway M, Sequeira E, Sherry ST, Sirotkin K, Souvorov A, Starchenko G, Tatusov RL (2008). Database resources of the National Center for Biotechnology Information. Nucleic Acids Res.

[B15] Lee Y, Sultana R, Pertea G, Cho J, Karamycheva S, Tsai J, Parvizi B, Cheung F, Antonescu V, White J, Holt I, Liang F, Quackenbush J (2002). Cross-referencing eukaryotic genomes: TIGR Orthologous Gene Alignments (TOGA). Genome Res.

[B16] Chen F, Mackey AJ, Stoeckert CJ, Roos DS (2006). OrthoMCL-DB: querying a comprehensive multi-species collection of ortholog groups. Nucleic Acids Res.

[B17] Iafrate AJ, Feuk L, Rivera MN, Listewnik ML, Donahoe PK, Qi Y, Scherer SW, Lee C (2004). Detection of large-scale variation in the human genome. Nat Genet.

[B18] Stamm S, Riethoven JJ, Le Texier V, Gopalakrishnan C, Kumanduri V, Tang Y, Barbosa-Morais NL, Thanaraj TA (2006). ASD: a bioinformatics resource on alternative splicing. Nucleic Acids Res.

[B19] Smigielski EM, Sirotkin K, Ward M, Sherry ST (2000). dbSNP: a database of single nucleotide polymorphisms. Nucleic Acids Res.

[B20] Lee TY, Huang HD, Hung JH, Huang HY, Yang YS, Wang TH (2006). dbPTM: an information repository of protein post-translational modification. Nucleic Acids Res.

[B21] Robinson J, Waller MJ, Stoehr P, Marsh SG (2005). IPD – the Immuno Polymorphism Database. Nucleic Acids Res.

